# PERSON-CENTERED CARE: DEFINITIONS AND PERCEPTIONS OF VARIOUS STAKEHOLDERS

**DOI:** 10.1093/geroni/igz038.1308

**Published:** 2019-11-08

**Authors:** Nancy Kusmaul, Gretchen Tucker

**Affiliations:** University of Maryland Baltimore County, Baltimore, Maryland, United States

## Abstract

Implementation of culture change in nursing homes shifts the care model from a traditional, more medically focused approach to person-directed care. Person-directed care promotes resident autonomy and decision making and the empowerment of direct care staff. In this paper, we examine how different stakeholders in nursing homes (residents, family members, direct care staff, administrative staff) conceptualize and experience a selection of person-centered care concepts (consistent assignment, meal choice, waking/bedtime practices, and bathing). We describe the commonalities and differences in the ways different groups of stakeholders operationalize these core person centered care practices and describe areas of potential conflict of views. Lastly, we consider how the well-being and quality of life for residents is affected by the use of these practices.

